# Diabetes Alters Activation and Repression of Pro- and Anti-Inflammatory Signaling Pathways in the Vasculature

**DOI:** 10.3389/fendo.2013.00068

**Published:** 2013-06-05

**Authors:** Elyse Di Marco, Stephen P. Gray, Karin Jandeleit-Dahm

**Affiliations:** ^1^Baker IDI Heart and Diabetes Research Institute, Melbourne, VIC, Australia; ^2^Department of Medicine, Monash University, Melbourne, VIC, Australia

**Keywords:** Nox, diabetes complications, atherosclerosis, immune cells, inflammation

## Abstract

A central mechanism driving vascular disease in diabetes is immune cell-mediated inflammation. In diabetes, enhanced oxidation and glycation of macromolecules, such as lipoproteins, insults the endothelium, and activates both innate and adaptive arms of the immune system by generating new antigens for presentation to adaptive immune cells. Chronic inflammation of the endothelium in diabetes leads to continuous infiltration and accumulation of leukocytes at sites of endothelial cell injury. We will describe the central role of the macrophage as a source of signaling molecules and damaging by-products which activate infiltrating lymphocytes in the tissue and contribute to the pro-oxidant and pro-inflammatory microenvironment. An important aspect to be considered is the diabetes-associated defects in the immune system, such as fewer or dysfunctional athero-protective leukocyte subsets in the diabetic lesion compared to non-diabetic lesions. This review will discuss the key pro-inflammatory signaling pathways responsible for leukocyte recruitment and activation in the injured vessel, with particular focus on pro- and anti-inflammatory pathways aberrantly activated or repressed in diabetes. We aim to describe the interaction between advanced glycation end products and their principle receptor RAGE, angiotensin II, and the Ang II type 1 receptor, in addition to reactive oxygen species (ROS) production by NADPH-oxidase enzymes that are relevant to vascular and immune cell function in the context of diabetic vasculopathy. Furthermore, we will touch on recent advances in epigenetic medicine that have revealed high glucose-mediated changes in the transcription of genes with known pro-inflammatory downstream targets. Finally, novel anti-atherosclerosis strategies that target the vascular immune interface will be explored; such as vaccination against modified low-density lipoprotein and pharmacological inhibition of ROS-producing enzymes.

## Introduction

A unifying feature of diabetic complications is chronic inflammation of the vasculature. The main vascular diseases that burden diabetic patients include nephropathy, retinopathy, neuropathy, and atherosclerosis. Each of these conditions has a significant immune component. In experimental and human diabetic nephropathy, infiltrating macrophages, and T cells elaborate a host of pro-inflammatory, pro-fibrotic, and pro-angiogenic factors that contribute to disease development and progression in the kidney (Lim and Tesch, 2012). Leukocyte adherence is causally associated with endothelial cell injury and cell death in the diabetic retina (Joussen et al., 2001) while infiltration of post-capillary venules with polymorphonuclear leukocytes is an early feature of proximal diabetic neuropathy (Kelkar et al., 2000). Similar to the microvasculature, immune-mediated inflammation of the macrovasculature plays a central role in the pathogenesis of diabetes-accelerated atherosclerosis (Libby et al., 2002). In this review, we examine the role of the immune response in the pathogenesis of atherosclerosis as a prototype for diabetes-associated vasculopathies.

## Triggers of Inflammation Elevated in Diabetes Compared to the Non-Diabetic Disease State

Atherosclerotic lesions represent an excessive inflammatory, fibro-proliferative response against different noxious stimuli (Ross, 1993, 1997). The diabetic milieu comprises of a host of potentially harmful, immunogenic products including modified forms of low-density lipoprotein (LDL), advanced glycation end products (AGEs), reactive oxygen species (ROS) as well as pro-inflammatory chemokines and cytokines. Elevated levels of LDL in patients with diabetes are subject to modification by both oxidation and glycation (Cohen et al., 2004). Furthermore, accelerated generation and vascular deposition of AGEs in addition to AGE interactions with RAGE in diabetes initiate oxidative reactions that promote the formation of oxidized LDL (oxLDL) (Basta et al., 2004). Oxidation of LDL within the sub-endothelial space (intima) enhances the pro-inflammatory properties of the endothelium (Mazière and Mazière, 2009) and activates both innate and adaptive arms of the immune system (Binder et al., 2002; Hansson et al., 2002).

## Relationship Between the Endothelium and Leukocytes in Early Atherogenesis

**Figure 1:**
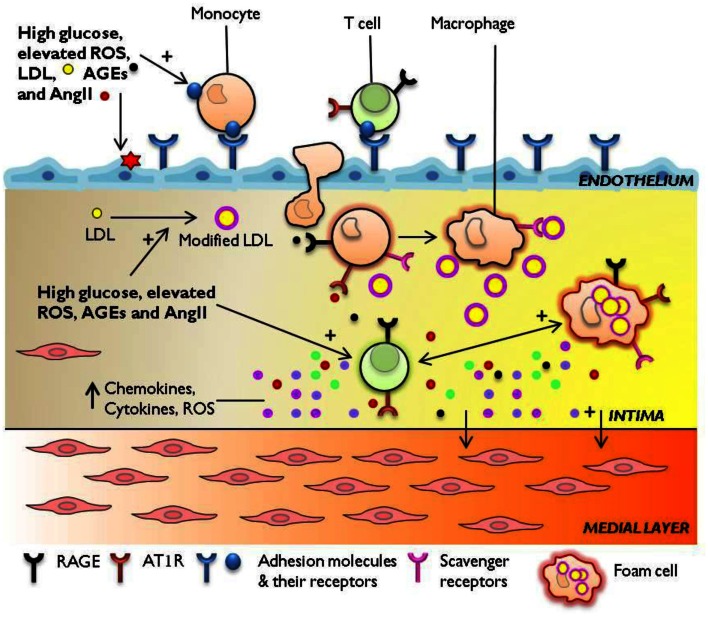
**Immune mechanisms engaged in diabetes-accelerated atherosclerosis**. Diabetes-associated hyperglycemia, hyperlipidemia, and oxidative stress render the endothelium dysfunctional, leading to the retention and oxidation of LDL molecules in the intimal space. The increased expression of adhesion molecules E-selectin, ICAM-1, VCAM-1 at the endothelial membrane, and upregulation of chemotactic molecules such as MCP-1 facilitate the continuous infiltration of immune cells to the inflamed aorta. Resident and monocyte-derived macrophages engulf LDL to form foam cells which release a host of pro-inflammatory cytokines, protease, and ROS. Activated T cells recruited from the circulation to the lesion also secrete cytokines which amplify pro-inflammatory cellular immune responses in the diabetic plaque. The diabetes-mediated increase in vascular inflammation drives the development and progression of atherosclerosis. AGEs, advanced glycation end-products; AT1R, angiotensin II type 1 receptor; ICAM-1, intercellular adhesion molecule-1; LDL, low-density lipoprotein; MCP-1, monocyte chemotactic protein-1; RAGE, receptor for advanced glycation end products; ROS, reactive oxygen species; VCAM-1, vascular cell adhesion molecule-1.

Chronic injury to the endothelium results in endothelial dysfunction, which can be defined as increased permeability, reduced nitric oxide (NO) dependent vasodilatation as well as enhanced pro-thrombotic and pro-inflammatory properties (Hink et al., 2001; Davignon and Ganz, 2004; Hartge et al., 2007). Endothelial dysfunction is a well established precursor of atherosclerosis, particularly in the setting of diabetes (Schalkwijk and Stehouwer, 2005). The diabetes-associated factors that impair normal endothelial function include increased synthesis of vasoconstrictors such as angiotensin II (Ang II) and endothelin-1, uncoupling of endothelial nitric oxide synthase (eNOS; leading to reduced bioavailability of NO), and increased expression and activity of ROS-producing enzymes (such as NADPH oxidases, Nox) which trigger the expression of adhesion and chemotactic molecules that promote the recruitment of inflammatory cells to the arterial wall (Figure 1) (Hink et al., 2001; Guzik et al., 2002; Lüscher et al., 2003; Hartge et al., 2007). Aortic lesions of diabetic *ApoE* knockout (KO) mice show increased gene expression of pro-inflammatory molecules MCP-1, VCAM-1, and NF-κB subunit p65 associated with increased pro-atherogenic cellularity [macrophages, T cells, and smooth muscle cells (SMCs)] compared to non-diabetic controls (Soro-Paavonen et al., 2008). In line with these *in vivo* findings, human endothelial cells exposed to high glucose conditions show increased leukocyte binding associated with increased expression of E-selectin, ICAM-1, VCAM-1, and MCP-1 through activation of NF-κB (Kim et al., 1994; Morigi et al., 1998; Piga et al., 2007). In addition, recent studies have revealed a striking association between glucose-induced endothelial expression of adhesion molecules VCAM-1 and P-selectin as well as the chemokines MCP-1 and fractalkine with Nox-derived ROS production (Manduteanu et al., 2010; Gray et al., 2013). These data indicate that diabetes-associated hyperglycemia and oxidative stress promote leukocyte-endothelial cell interactions required for the recruitment of leukocytes to the inflamed vessel.

## Role of Smooth Muscle Cells in Diabetes-Mediated Vascular Inflammation

Vascular SMCs play a central role in the initiation and progression of atherosclerosis (Doran et al., 2008). The expression of cellular adhesion molecules and pro-inflammatory cytokines by lesional SMCs promote the accumulation and activation of leukocytes in the atherosclerotic lesion (Braun et al., 1999; Raines and Ferri, 2005). Of note, the expression of VCAM-1, ICAM-1, and fractalkine is confined to SMCs within atherosclerotic vessels (or lesion-prone areas) but not in healthy vessels (O’Brien et al., 1993; Endres et al., 1997; Barlic et al., 2007); providing further evidence of the pro-atherogenic role of these molecules. In response to diabetes-associated atherogenic stimuli, such as Ang II (Ruiz-Ortega et al., 2003) and ROS (Su et al., 2001), SMCs elaborate an array of extracellular matrix proteins which facilitate the retention of inflammatory cells and stabilization of the plaque including the formation of a fibrous cap. Diabetes stimulates and sustains the inflammatory “synthetic” phenotype of vascular SMCs. Hyperglycemia enhances the migration and proliferation of resident SMCs within the intimal lesion which contributes to the accelerated formation of advanced atherosclerosis in diabetes (Suzuki et al., 2001). Furthermore, exposure of cultured SMCs to high glucose or AGEs significantly increases the production of ROS by Nox enzymes (Inoguchi et al., 2000; Wautier et al., 2001; Gray et al., 2013). More specifically, recent evidence has identified a central role for the Nox1 isoform in glucose (Gray et al., 2013) and Ang II-induced vascular superoxide production (Lassègue et al., 2001). Therefore, vascular SMCs represent key players in diabetes-mediated pro-inflammatory and pro-oxidant responses that drive atherosclerosis.

## Macrophages are a Source of Pro-Oxidant, Pro-Inflammatory Molecules Which Fuel Pro-Atherogenic Processes in the Vessel Wall

The macrophage plays a critical role in the initiation and progression of the atherosclerotic lesion. Circulating monocytes are recruited to inflamed regions of the endothelium where they engulf trapped LDL to form foam cells, histologically visible as the early fatty streak (Ross, 1997). Uptake of glycated LDL by human monocyte-derived macrophages occurs to a greater extent than for native LDL (Klein et al., 1995). Interestingly, glycoxidized LDL increases scavenger receptor expression to a greater extent than glycated LDL or oxLDL alone (Lam et al., 2004). Therefore, hyperglycemia in combination with elevated ROS in diabetes increases macrophage avidity for LDL, and thus foam cell formation, which ultimately activates other macrophages, infiltrating adaptive “effector” cells as well as resident vascular cells leading to accelerated atherosclerosis.

The lipid-laden macrophage acts as a frustrated phagocyte, leaking ROS (refer to Nox section), and metalloproteases (MMPs) into the extracellular space which catalyze the degradation of the extracellular matrix proteins and trigger apoptosis of SMCs that support the plaque’s fibrotic cap, ultimately enhancing the risk of plaque rupture (Shah et al., 1995). Macrophages function in response to environmental cues (Waldo et al., 2008). Monocyte-derived macrophages cultured in high glucose (25 mM) conditions display increased expression and activity of MMP-9 without affecting metalloproteinase inhibitor (TIMP-1) expression (Death et al., 2003). In line with these findings, Devaraj et al. (2006) showed that monocyte superoxide anion release, pro-inflammatory cytokines interleukin (IL) 6, and IL1β were significantly elevated in type 1 diabetic subjects compared with in control subjects. These striking effects of hyperglycemia on macrophage activity may provide some insight into the significantly increased incidence of plaque rupture, thrombosis, and ultimately myocardial infarction in diabetic patients compared to their non-diabetic counterparts (Silva et al., 1995).

## Interactions Between Leukocytes Within the Lesion Engage Inflammatory Pathways that Drive Atherosclerosis

Monocyte-derived macrophages act as sentinels of pro-inflammatory signals, able to be activated by diverse stimuli and release a range of mediators that contribute to pro- as well as anti-atherogenic processes (Moore and Tabas, 2011). A plethora of cytokines are detected in atherosclerotic vessels, and the reader is directed to excellent reviews by Tedgui and Mallat (2006) and Ait-Oufella et al. (2011). Activated macrophages and foam cells release a number of pro-inflammatory cytokines and chemokines which facilitate the continual recruitment of monocytes and T lymphocytes to the atherosclerotic lesion. Of note, the predominance of IFNγ-producing T lymphocytes in the lesion is likely to contribute significantly to local macrophage activation (de Boer et al., 1999). Further gene expression analysis of advanced human plaques has revealed a cytokine profile indicative of a pro-inflammatory, Th1-type cellular immune response (Frostegård et al., 1999). The increased expression of CD40 and MHC-II molecules on plaque macrophages (and other plaque-associated vascular cells) also suggests an enhanced capacity of these cells to function as antigen-presenting cells to T lymphocytes (Hansson et al., 1991; Li et al., 1993; Mach et al., 1997). The potent immunostimulatory and pro-atherogenic effects of IFNγ are well established (Gupta et al., 1997), and so interactions between macrophages and T lymphocytes that continuously upregulate IFNγ production are expected to contribute to the progression of the lesion.

## The Cytokine Profile of Activated Vascular Cells/Leukocytes is Altered in the Diabetic Setting

Macrophages represent a heterogeneous population of distinct subsets capable of differentially affecting Th1- or Th2-type responses (Mills et al., 2000). Lesion macrophages adopt predominantly a pro-inflammatory and pro-atherogenic role indicative of the M1-subtype (Khallou-Laschet et al., 2010; Wilson, 2010; Shalhoub et al., 2011). Furthermore, hyperglycemia interferes with the ability of IL4 to polarize macrophages to the alternatively activated M2 state, characterized by wound repair and anti-inflammatory functions (Auffray et al., 2009; Parathath et al., 2011). A comprehensive review of macrophage differentiation and activation pathways in atherosclerosis can be found by Shalhoub et al. (2011). In a similar manner, hyperglycemia is associated with increased activation of pro-inflammatory human T lymphocytes and decreased suppressor function of T regulatory cell subsets (Marfella et al., 2003; Lindley et al., 2005; Stentz and Kitabchi, 2005). Therefore, not only does the diabetic micro-environment amplify the magnitude and array of new, potentially immunogenic stimuli but it also skews the leukocyte repertoire in favor of adopting a pro-inflammatory functional phenotype. Moreover, high glucose may interfere with the immunomodulatory capacity of T cell and macrophage subsets leading to an unabated prolongation of the inflammatory process.

Patients with diabetes have an increased risk of infection attributable to defects in both innate and adaptive immunity (Geerlings and Hoepelman, 1999). Consistent with these findings in humans, streptozotocin (STZ)-induced diabetes in rodents predisposes to delayed innate (Chin et al., 2012) and adaptive (Vallerskog et al., 2010) responses irrespective of the challenge, leading to increased susceptibility to infection. High glucose conditions have been shown to decrease T and B lymphocyte proliferation, diminish cell viability, and increase apoptosis via mechanisms likely to involve increased oxidative stress (Rubinstein et al., 2008). This seems counter-intuitive in a disease characterized by secondary complications driven by immune cell-mediated inflammation, however the high glucose and high ROS diabetic milieu provides the ideal setting for chronic activation of innate and adaptive cells to non-microbial, modified macromolecules (described in section above). Therefore, it is important to discuss diabetes-induced alterations in the immune system in the context of any insults (infection) or complications (e.g., atherosclerosis) that accentuate as well as catalyze further derangement in both arms of the immune response.

## Signaling Pathways Important to Hyperglycemia-Induced Vascular Pathophysiology

### AGE/RAGE

Advanced glycation end products and their cell surface receptor, RAGE, have been implicated in the amplification and progression of immune-inflammatory responses that underscore diabetic complications (Schmidt et al., 2001; Basta et al., 2004; Jandeleit-Dahm et al., 2008; Bierhaus and Nawroth, 2009). Atherosclerotic lesions in diabetic *ApoE* KO mice display increased accumulation of AGEs and enhanced expression of RAGE (Kislinger et al., 2001; Wendt et al., 2002; Soro-Paavonen et al., 2008). Ligation of RAGE by AGE stimulates endothelial pro-inflammatory gene expression (VCAM-1, E-selectin) and ROS production in a NADPH-oxidase dependent manner (Schmidt et al., 1995; Wautier et al., 2001; Higai et al., 2006). In macrophages, AGE–RAGE interaction prompts cell chemotaxis and cytokine release via a mechanism involving the activation of NADPH oxidase (Kirstein et al., 1990; Wautier et al., 2001). RAGE also appears to play a role in T lymphocyte activation and differentiation, mediating Th1-type responses such as the synthesis of IFNγ (Chen et al., 2008). Recent work by Akirav et al. (2012) found that RAGE is expressed intracellularly in human T cells following TCR activation but constitutively on T cells from patients with diabetes. The current evidence supports a critical role of AGE/RAGE signaling in the chronic activation of the immune-inflammatory processes that accelerate atherosclerosis in diabetes.

### Ang II/AT1R

The renin-angiotensin system (RAS) plays an important role in the regulation of vascular contractility and also represents a primary target for diabetes-induced vascular dysfunction and inflammation. Most of the known pathophysiological effects of Ang II are mediated via the activation of the Ang II type 1 (AT1) receptor. The production of Ang II and the expression of its AT1R are upregulated in the aorta of diabetic mice (Candido et al., 2002, 2004). Signaling via the AT1R has been shown to upregulate the RAGE pathway in diabetic atherosclerosis (Ihara et al., 2007). Furthermore, Ang II induces activation with increased adhesion molecule expression in endothelial cells, monocytes, and T lymphocytes (Hahn, 1994; Tummala et al., 1999; Hoch et al., 2009). Most of the signaling events secondary to Ang II-AT1R binding are redox sensitive, such as NFκB activation, and rely on the production of ROS by Nox enzymes (Pueyo et al., 2000; Alvarez and Sanz, 2001; Liu et al., 2003). Treatment of diabetic mice with an AT1R blocker such as Candesartan attenuated ROS production and Nox activity leading to improvements in endothelial function (Oak and Cai, 2007). Recent work by Valente et al. (2012) revealed a direct physical association between the AT1R and the Nox1-NADPH-oxidase isoform in vascular SMCs, however the potential functional and regulatory implications of this relationship remain to be explored in the context of hyperglycemia. A common feature of AGE and Ang II signaling is the convergence on Nox-derived ROS as secondary mediator molecules required to elicit the effects of RAGE and AT1 receptor binding, respectively.

### NOX/ROS

Hyperglycemia induces activation of the Nox family NADPH-oxidase enzymes and the consequent ROS production contributes to the pathophysiological complications of diabetes (Gao and Mann, 2009). Nox enzymes are the major source of ROS in the vessel wall, differentially expressed in vascular and infiltrating inflammatory cells (Griendling and Ushio-Fukai, 1997; Lassègue and Clempus, 2003). In particular, the isoforms Nox1, Nox2, Nox4, and Nox5 play important roles in a wide range of physiological and pathological processes relevant to cardiovascular disease (Sorescu et al., 2002; Bedard and Krause, 2007; Lassègue and Griendling, 2010). Increased ROS production in the diabetic aorta positively correlates with upregulated expression and activity of Nox1, Nox2, and Nox4 (Guzik et al., 2002; Wendt et al., 2005; Hwang et al., 2007). Nox1 KO mice are protected from endothelial dysfunction in diabetes (Youn et al., 2012) while studies in Nox4 KO mice have reported a vasculo-protective role for endogenous Nox4 (Schröder et al., 2012). Furthermore, recent work by Gray et al. (2013) in Nox1 and Nox4/*ApoE* DKO mice identified a key role for Nox1 but not Nox4-derived ROS in diabetes-accelerated atherosclerosis which is consistent with previous reports suggesting different (patho)physiological functions for Nox isoforms in the vasculature.

Reactive oxygen species are both a product, and modulator, of leukocyte recruitment, activation, and function. Ligation of VCAM by leukocytes activates endothelial NADPH oxidase which results in enhanced ROS production (Deem and Cook-Mills, 2004). In macrophages, Nox2 (gp91phox) is the major source of ROS contributing to the respiratory burst, while Nox4 is inducible by oxLDL (Lee et al., 2010; Tavakoli and Asmis, 2012). T lymphocytes also express a phagocyte-type Nox that is activated after TCR stimulation (Jackson et al., 2004). Further activation of vascular Nox by NFκB-dependent pro-inflammatory cytokines, including TNFα, reaffirms the relationship between oxidative stress and immune-mediated inflammation in diabetes (Park et al., 2006; Gauss et al., 2007; Miller et al., 2007). The potential impact of diabetes-mediated changes in Nox on ROS-sensitive leukocyte functions represents a relatively unexplored area of investigation.

## Athero-Protective Mechanisms: Focus on Antioxidants

We have discussed some of the key pro-inflammatory, pro-atherosclerotic effects of Ang II/AT1R and AGE/RAGE signaling pathways that are aberrantly activated in response to hyperglycemia. However, it is important to note that these same pathways can elicit anti-inflammatory effects, and the reader is encouraged to consult Goh and Cooper (2008), Daugherty et al. (2010), and Thomas et al. (2010) for detail on alternative AGE receptors, AT2R, and ACE2. While pro-inflammatory signaling cascades tend to predominate in diabetes, the inactivation of some important athero-protective pathways by the diabetic milieu further exposes the diabetic vasculature to pro-oxidant insults that accelerate atherogenesis. Major anti-oxidant defense systems such as superoxide dismutases (SODs), and glutathione peroxide 1 (Gpx1) are critical in the maintenance of redox balance in the intracellular and extracellular spaces. Diabetic *ApoE/GPx1* DKO mice show significantly more aortic atherosclerosis associated with enhanced macrophage recruitment, RAGE, and VCAM-1 expression compared to the diabetic *ApoE* KO mice (Lewis et al., 2007). Therefore, a decrease in anti-oxidant capacity in diabetes in combination with elevated ROS production from various sources, contributes to the pro-oxidant diabetic milieu (Feillet-Coudray et al., 1999; Vessby et al., 2002).

## An Emerging Role for Epigenetic Modifications in Diabetic Complications

The effect of hyperglycemia on the transcription and translation of genes coding proteins involved in the inflammatory response persist beyond the time of exposure. A growing number of studies utilizing the bio-informatic power of epigenetic medicine have already identified chromatic modifications characterized by histone methylation and acetylation sites, and their associated enzymes, involved in glucose-mediated changes in gene expression (Brasacchio et al., 2009; Pirola et al., 2011; Keating and El-Osta, 2012; Miao et al., 2012). El-Osta et al., identified a role for Set7, a H3K4-specific methyltransferase, in transient hyperglycemia-mediated chromatin changes at the promoter of the NF-κB subunit p65 resulting in increased gene expression of p65, MCP-1, and VCAM-1 in vascular endothelial cells that persist during subsequent normoglycemia; a phenomenon termed “glycemic memory” (El-Osta et al., 2008; Okabe et al., 2012). Similarly, *ex vivo* culture of vascular SMCs from type 2 diabetic *db/db* mice exhibit a sustained atherogenic and pro-inflammatory phenotype with concomitant depletion of the H3K9-specific methyltransferase, Suv39h1, despite restoration of euglycaemia (Villeneuve et al., 2010). Further investigations suggest that regulation of pro-inflammatory gene expression by Suv39h1 is dependent on glucose-mediated elevations in the microRNA (miR)-125b (Villeneuve et al., 2008).

Recent advances in cancer research have demonstrated cross-talk between miR machinery and DNA methylation (Ting et al., 2008; Yan et al., 2011). While there is growing evidence supporting glucose-induced alterations in chromatin structure and miR expression in the pathogenesis of diabetic complications (Reddy and Natarajan, 2011; Villeneuve et al., 2011), little is known about the interplay of these components in diabetes.

Epigenetic mechanisms play a central role in the pathobiology of endothelial (Piconi et al., 2004; El-Osta et al., 2008) and SMCs (Villeneuve et al., 2008, 2010) in high glucose conditions, yet remain a relatively unexplored area in the context of immune cell-mediated vascular inflammation. Monocytes and lymphocytes, immune cells with well-characterized roles in atherosclerosis (Hansson and Hermansson, 2011), display distinct profiles of histone acetylation and methylation in diabetic patients with corresponding changes in inflammatory gene expression (Miao et al., 2007, 2008). Additionally, Miao et al. (2012) identified marked differences in human leukocyte antigen (HLA)-expression in monocytes from diabetic patients compared to controls which related to differences in the histone acetylation status of the HLA promoter region. In light of these findings, it would be very interesting to explore the epigenetic status of various immune cell populations, especially adaptive immune cells which naturally develop “memory” subsets [present also in complex atherosclerotic lesions (Stemme et al., 1992)] that respond rapidly to repeated challenges of the same antigen. Poorly controlled diabetes whereby blood glucose regularly spikes to pathological levels may be sufficient to reactivate memory subpopulations, leading to rapid and robust inflammatory responses that accelerate atherosclerosis and increase risk of plaque rupture events in diabetes. Future studies examining chromatin modifications in immune cells intimately involved in the pathogenesis of diabetic complications will improve our understanding of the mechanisms underlying glycemic memory and the sustained pro-inflammatory state of the diabetic vasculature.

## Therapeutic Strategies with Clinical Promise

Therapeutic strategies against diabetes-associated atherosclerosis can be targeted at various stages of disease pathogenesis. Interventions that block signaling via RAGE (Soro-Paavonen et al., 2008) or the AT1R (Candido et al., 2004) have proved effective in reducing plaque formation in mouse models with diabetes. There is a growing body of evidence to suggest that a large number of the pro-inflammatory signaling cascades triggered by hyperglycemia converge on Nox-derived ROS making it an excellent target for novel therapies (Sedeek et al., 2012). To date, studies examining pharmacological inhibition of Nox-derived ROS in experimental models of chronic inflammatory diseases including liver fibrosis (Jiang et al., 2012), diabetic nephropathy (Sedeek et al., 2010), and atherosclerosis (Gray et al., 2013) have yielded positive results, and much anticipation surrounds the clinical evaluation of specific Nox inhibitors (Kim et al., 2011). The effects of immune modulation on atherosclerosis and the emerging inflammatory cell/cytokine-directed therapies have been addressed in a recent review by Little et al. (2011). Increasing evidence demonstrates a strong association between circulating oxLDL-immune complexes and cardiovascular risk in diabetic patients (Lopes-Virella et al., 1999, 2011a,b; Orchard et al., 1999). Immunization with oxLDL or other candidate plaque antigens have shown promise in animal models (Palinski et al., 1995; Ameli et al., 1996; George et al., 1998) however evidence demonstrating the pathogenic properties of antibodies to modified LDL (summarized by Lopes-Virella and Virella, 2010) may hamper the clinical utility of this strategy. Cytokine-based therapies that skew macrophage or T cell to anti-inflammatory and regulatory phenotypes have been effective in mouse models of atherosclerosis (Namiki et al., 2004; Sasaki et al., 2009; Cardilo-Reis et al., 2012). The establishment of highly atherosclerosis-specific antigens or cytokines in combination with cell- or tissue-focused delivery systems will be pre-requisites for more fine-tuned immunomodulatory therapies. Overall, significant challenges continue to plague attempts to translate laboratory findings to the clinic and greater emphasis on human studies is required to fully realize the therapeutic potential of targeting immunological mechanisms in disease (Davis, 2008; Libby et al., 2011). A systems approach to immunology, as outlined in a recent review by Brodin et al. (2013), may impart more successful clinical results for novel therapies.

## Conclusion

An extensive body of experimental and clinical evidence has improved our understanding of the immune-inflammatory processes involved in vascular disease. However, there are still considerable challenges in alleviating the burden of vascular complications in diabetic patients. In this review we have discussed that, in addition to high glucose, pathological increases in AGEs, Ang II, and ROS induce a chronic state of vascular inflammation. Specifically, we have described how diabetes-mediated aberrations in vascular-leukocyte interactions result in increased accumulation of pro-inflammatory leukocytes to the atherosclerotic vessel wall. Future studies that explore the pro-inflammatory effects of RAGE, AT1R, or Nox-signaling pathways in the diabetic vasculature are likely to offer novel ways of targeting the immune responses inappropriately activated or inactivated in diabetes.

## Conflict of Interest Statement

The authors declare that the research was conducted in the absence of any commercial or financial relationships that could be construed as a potential conflict of interest.
